# Rewards Enhance Proactive and Reactive Control in Adolescence and Adulthood

**DOI:** 10.1093/scan/nsz093

**Published:** 2019-12-10

**Authors:** Lucía Magis-Weinberg, Ruud Custers, Iroise Dumontheil

**Affiliations:** 1 Department of Experimental Psychology, University College London, London WC1H 0AP, UK; 2 Institute of Human Development, University of California, Berkeley 94720 Berkeley, USA; 3 Department of Psychology, Utrecht University, 3584 CS Utrecht, The Netherlands; 4 Department of Psychological Sciences, Birkbeck, University of London, London WC1E 7HX, UK

**Keywords:** cognitive control, development, reward, adolescence, fMRI

## Abstract

Cognitive control allows the coordination of cognitive processes to achieve goals. Control may be sustained in anticipation of goal-relevant cues (proactive control) or transient in response to the cues themselves (reactive control). Adolescents typically exhibit a more reactive pattern than adults in the absence of incentives. We investigated how reward modulates cognitive control engagement in a letter-array working memory (WM) task in 30 adolescents (12–17 years) and 20 adults (23–30 years) using a mixed block- and event-related functional magnetic resonance imaging design. After a Baseline run without rewards, participants performed a Reward run where 50% trials were monetarily rewarded. Accuracy and reaction time (RT) differences between Reward and Baseline runs indicated engagement of proactive control, which was associated with increased sustained activity in the bilateral anterior insula (AI), right dorsolateral prefrontal cortex (PFC) and right posterior parietal cortex (PPC). RT differences between Reward and No reward trials of the Reward run suggested additional reactive engagement of cognitive control, accompanied with transient activation in bilateral AI, lateral PFC, PPC, supplementary motor area, anterior cingulate cortex, putamen and caudate. Despite behavioural and neural differences during Baseline WM task performance, adolescents and adults showed similar modulations of proactive and reactive control by reward.

## Introduction

Adolescents’ ability to exert cognitive control is particularly susceptible to potential rewards and affectively charged contexts (Crone and Dahl, 2012; Cohen *et al.,* 2016; van Duijvenvoorde *et al.,* 2016). Prevailing frameworks suggesting a maturational imbalance in adolescence have focused on instances when cognitive control fails to constrain reward-sensitive systems, leading to potentially negative outcomes, typically during risky decision-making (Casey, 2015). Less is known about situations in which cognitive control might be enhanced by reward sensitivity (Strang and Pollak, 2014). In this study, we explored whether adolescents and adults can adaptively engage cognitive control processes as a function of the temporal dynamics of reward to maximise their performance in a working memory (WM) task.

The dual mechanisms of control (DMC) framework distinguishes between two temporally distinct cognitive control strategies (Braver, 2012). Proactive control refers to the sustained maintenance of goal-relevant information in anticipation of a cue. Reactive control refers to the transient reactivation of goals in response to a cue. Reactive control is less demanding than proactive control but more susceptible to interference (Braver, 2012; Chiew and Braver, 2017). While adults vary in the recruitment of proactive and reactive control as a function of trait factors (Locke and Braver, 2008; Chiew and Braver, 2017), they can flexibly engage the most efficient mode of cognitive control to adapt to contextual demands, as evidenced by changes in response to experimental manipulations (Braver *et al.,* 2009; Chiew and Braver, 2013). Mixed event-related/blocked functional magnetic resonance imaging (fMRI) designs (Visscher *et al.,* 2003) are specifically optimised to dissociate sustained *vs* transient changes in neural activation within a single experimental paradigm. These designs have been employed to study cognitive control (Marklund *et al.,* 2007; Brahmbhatt *et al.,* 2010; McDaniel *et al.,* 2013) and the impact of reward manipulations on cognitive control strategies (Jimura *et al.,* 2010). fMRI studies have predominantly implicated the frontoparietal network in implementing proactive and reactive control (Braver *et al.,* 2009; Jimura *et al.,* 2010). In addition, the dorsal anterior cingulate cortex (ACC) is also involved in sustained control, and the rostral ACC is involved in reactive compensations (Jiang *et al.,* 2015). Further, the anterior insula (AI) participates in estimating the volatility of control demands and the caudate in predicting forthcoming demands (Jiang *et al.,* 2015).

Reliance on reactive control in early childhood shifts towards a mix of proactive and reactive control depending on individual differences and task demands in mid- to late childhood (Chevalier *et al.,* 2015). By age 8, children seem to have the capacity to flexibly adapt strategies to be more efficient (Chatham *et al.,* 2009; Blackwell and Munakata, 2014; Chevalier *et al.,* 2015). In a handful of fMRI studies, more protracted proactive control development compared to reactive control has been described in adolescence (Velanova *et al.,* 2009; Andrews-Hanna *et al.,* 2011; Alahyane *et al.,* 2014) and was associated with reduced prefrontal activity in adolescents compared to adults in posterior dorsolateral prefrontal cortex (PFC) (Andrews-Hanna *et al.,* 2011) and with reduced sustained activity in children and adolescents compared to adults in a region near the inferior frontal junction (Velanova *et al.,* 2009). In contrast, Alahyane *et al.* (2014) found that adolescents and adults had comparable frontoparietal activity associated with prosaccade and antisaccade preparation, which was higher than in children (8–12 years old).

The balance between reactive and proactive cognitive control is sensitive to the motivational context (Braver *et al.,* 2014; Chiew and Braver, 2017) and interacts with reward circuitry in the presence of incentives (Luna *et al.,* 2015). Reward-driven enhancement of performance may be driven by top-down control mechanisms that modulate the processing of subsequent stimuli in preparatory fashion through increased sustained proactive control (Locke and Braver, 2008; Jimura *et al.,* 2010) or transient increases in reactive control on a trial-by-trial basis (Jimura *et al.,* 2010). There is also evidence for some contribution by more automatic bottom-up processes, suggesting increased saliency of reward-related features (Krebs *et al.,* 2015).

In the absence of reward, cognitive control continues to develop and become more stable during adolescence (for WM, see review in Zanolie and Crone, 2018). Over the course of development, cognitive control-related prefrontal activation becomes more attuned to varying contextual demands (Chevalier *et al.,* 2019). Adolescents can improve their inhibitory control performance to match adults’ performance in the presence of reward (Geier *et al.,* 2010; Padmanabhan *et al.,* 2011; Luna *et al.,* 2015; Zhai *et al.,* 2015). Along these changes in performance, in the reward context, adolescents show increased transient recruitment of cognitive control regions (frontal cortex along the precentral sulcus) and reward regions (ventral striatum) during response preparation, compared to adults (Geier *et al.,* 2010; Padmanabhan *et al.,* 2011). Corticostriatal coupling under high and low rewards continues to develop in adolescence, underlying the increased capacity of adults to modulate cognitive control selectively in the context of high rewards (Insel *et al.,* 2017). Overall, this suggests greater integration of executive control and motivation during development (Smith *et al.,* 2014).

In contrast, Strang and Pollack (2014) found that in a task of proactive and reactive control [AX-Continuous Performance Test] children, adolescents and adults between 9 and 30 years old showed a similar ability to shift into a proactive control strategy in the context of reward, associated with increased sustained activity in the right lateral PFC, right posterior parietal cortex (PPC) and right AI, among other regions. An outstanding question is whether modulation of transient brain activity can also be observed across age groups. Greater modulation of prefrontal activation in response to contextual demands has been proposed as one of the developmental mechanisms underlying cognitive control development (Chevalier *et al.,* 2019).

Here, we investigated the age-related increases in proactive and reactive cognitive control and their modulation by a motivational (reward) context that varied trial-by-trial and across blocks. We employed a mixed block- and event-related fMRI design while adolescents and adults completed a WM task in neutral and reward conditions adapted from Jimura *et al.* (2010). We used a mixed experimental design which allowed us to detect sustained brain activity across blocks (proactive control) and transient activity in response to trials (reactive control). We expected adolescents to be more reliant on reactive control and to show greater sensitivity to a rewarding context, in terms of behaviour and transient neural activity, while we expected adults to exhibit a more proactive control strategy, with associated sustained frontoparietal activity across blocks.

## Methods

### Participants

Thirty adolescents (15 females, 12–17 years old, mean [*M*] = 14.6 ± 1.4 [standard deviation (*SD*)]) and 20 adults (10 females, 22–30 years old, *M* = 27.1 ± 1.9) participants took part in this study. Participants were reimbursed £20 (plus up to £8 depending on their performance on the task) and their travel expenses. This study was approved by the University College London Research Ethics Committee. Consent was obtained according to the Declaration of Helsinki, adults and the parents of adolescents provided written consent while adolescent themselves gave verbal consent. Adolescent and adult groups did not differ in their age-normed scores on the Vocabulary subtest of the Wechsler Abbreviated Scale of Intelligence (WASI-II; Wechsler, 2011) [adolescents: *M =* 66.9 ± 0.9 (s.d.); adults: *M =* 64.7 ± 1.7; *t*(28.8) = 1.15, *P* = 0.25].

### Experimental design and stimulus material

#### Design

**Fig. 1:**
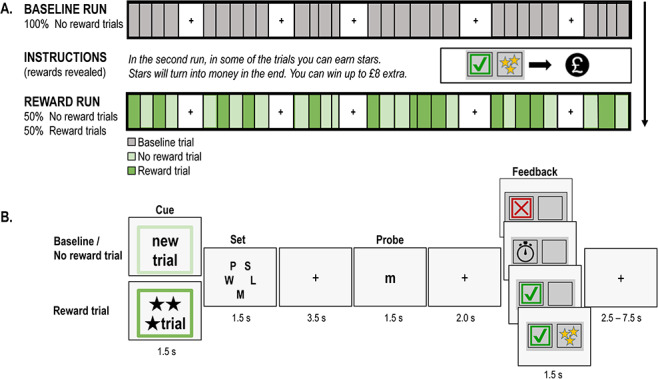
(Colour online) Letter-array task. (A) Experimental design. Participants performed two runs of 30 trials each of a letter-array WM task. In the first run, none of the trials was rewarded. In the second run, half of the trials could be rewarded. (B) Example trials. On each trial, participants were presented with a five-letter set and, after a delay, had to indicate whether the probe was present in the set. Each trial was preceded with a cue screen indicating whether participants could earn rewards (stars) on this trial and followed by feedback on performance. The next trial started after an intertrial interval lasting 2.5, 5 or 7.5 s.

The fMRI task had one between-subjects factor (age group, adults and adolescents) and two types of within-subject factors: either sustained, run effects (*Baseline run vs Reward run*) or transient, trial effects (*Baseline trials vs Reward trials vs No reward trials*). In the *Reward run*, half of the trials had potential rewards (*Reward trials*) and half did not (*No reward trials*). Preceding the *Reward run*, participants were unaware of the potential rewards, and hence all the *Baseline trials* in the *Baseline run* were unrewarded (Fig. 1A).

#### Letter-array WM task

We employed a fixed set-size Sternberg-item recognition task adapted from Jimura, Locke and Braver (2008) (Fig. 1B). At the beginning of each WM trial, a cue indicated whether a potential reward could be obtained on this trial (*Reward trial*) or not (*Baseline trial* or *No reward trial*). Five uppercase consonants were then presented and after a retention interval a single lowercase probe letter. Participants indicated by pressing one of two buttons on a handheld response box whether the probe matched one of the letters from the memory set (right index finger) or not (right middle finger). Participants were encouraged to respond both accurately and quickly. Visual feedback indicated whether the response was incorrect, too slow, correct and not rewarded or correct and rewarded (Fig. 1B). Cut-off times were individually set for each participant based on his/her own median correct reaction time (RT) on trials performed in the practice.

#### Other behavioural measures

After scanning, participants completed computerised versions of the Behavioural Activation and Inhibition Scale (BIS/BAS; Carver and White, 1994); Sensitivity to Punishment and Sensitivity to Reward Questionnaire (SPSRQ; Torrubia *et al.,* 2001); WEBEXEC, a web-based short self-report of executive functions (Buchanan *et al.,* 2010); and a simple Go/No Go task (Simmonds *et al.,* 2008; Humphrey and Dumontheil, 2016). Lastly, participants completed forward and backward digit span tasks and the Vocabulary subtest of the WASI-II. In addition, after scanning, participants rated how rewarding they found both stars and money on a scale from 1 (not at all) to 5 (very rewarding).

### Procedure

Participants were trained on the letter-array WM task outside the scanner. After receiving task instructions, participants performed one block of 10 trials with a cut-off time of 2.5 s and one further block of 15 trials with their individual cut-off time limit (median RT in the first 10 trials). This was done to adapt task difficulty to each individual and quickly achieve a consistent level of performance.

In the scanner, participants first performed the Baseline run (30 Baseline trials) of the task. At this point, participants were naïve regarding the chance to earn further money based on their performance on the task. Participants were then given further instructions regarding the reward component of the second run; they were told: ‘In the second run, in some of the trials you can earn stars. Stars will turn into money in the end. You can win up to £8.00’. Participants were also introduced to the reward cues and reward feedback. Participants then performed the Reward run (15 Reward trials, 15 No reward trials). The order of the trial types was fixed in one of two possible sequences, which were counterbalanced across participants. Sequences started with the presentation of a Reward trial and did not present the same trial type more than twice in a row (i.e. RNRRN RNRN RNRNN NRNRRN). Task blocks lasted 57.5–95.0 s and alternated with fixation periods lasting 21.9–29.7 s. Block starts and ends were indicated by a 1.5 s instruction screen. The task was programmed in Cogent (www.vislab.ucl.ac.uk/cogent_graphics.php) running in MATLAB (The MathWorks, Inc., Natick, MA).

### Image acquisition

Functional data were acquired using The Center for Magnetic Resonance Research (CMRR) at the University of Minnesota multiband echo-planar imaging sequence (Xu *et al.,* 2013) 2x acceleration, leak block on (Cauley *et al.,* 2014) with blood-oxygen-level-dependent (BOLD) contrast (44 axial slices with a voxel resolution of 3 × 3 × 3 mm covering most of the cerebrum; repetition time (TR) = 2 s; echo time (TE) = 45 ms; acquisition time (TA) = 2 s) in a 1.5 T MRI scanner with a 30-channel head coil (Siemens TIM Avanto, Erlangen, Germany). Participants completed two scanning runs in which 321 functional volumes were obtained. A T1-weighted Magnetization Prepared - RApid Gradient Echo (MPRAGE) with 2× GeneRalized Autocalibrating Partial Parallel Acquisition (GRAPPA) acceleration anatomical image lasting 5 min 30 s was acquired after the functional runs.

### Data analysis

#### Behavioural data analysis

2 (age group) × 3 (trial type: Baseline, No reward, Reward) mixed-model repeated measures analysis of variances (ANOVAs) were performed on correct trials mean RT and accuracy of the letter-array WM task. Models were fitted in R 3.5.2 (R Core Team, 2018) using afex (Singmann *et al.,* 2018). Greenhouse–Geisser correction was employed for violation of sphericity and Tukey correction for multiple comparisons.

#### MRI data pre-processing

MRI data were pre-processed and analysed using Statistical Parametric Mapping (SPM12, Wellcome Trust Centre for Neuroimaging, http://www.fil.ion.ucl.ac.uk/spm/). Images were realigned to the first analysed volume with a second-degree B-spline interpolation. The bias-field corrected structural image was coregistered to the mean, realigned functional image and segmented using Montreal Neurological Institute (MNI)-registered International Consortium for Brain Mapping tissue probability maps. Resulting spatial normalisation, parameters were applied to obtain normalised functional images with a voxel size of 3 × 3 × 3 mm, which were smoothed with an 8 mm full width at half maximum Gaussian kernel. Realignment estimates were used to calculate framewise displacement (FD) for each volume (Siegel *et al.,* 2014). Volumes with an FD >0.9 mm were censored and excluded from general linear model estimation by including a regressor of no interest for each censored volume. Adolescents and adults did not differ in estimated movements (all *P*’s >0.28) except adolescents had a lower root mean square translational movement (*M* = 0.18, s.d. = 0.07) than adults (*M* = 0.24, s.d. = 0.12, *P* = 0.03).

#### Block- and event-related fMRI data analysis

Sustained activity was modelled in Reward and No reward runs separately using extended boxcar regressors representing task and fixation blocks. Transient activity was modelled using two boxcar regressors of 10.5 s, representing correctly answered Reward trials and No reward trials (in the Baseline run, this distinction was arbitrary but matched the order of Reward and No reward trials in the Reward run). Other regressors were start of blocks (1.5 s), end of blocks (1.5 s), incorrect trials (10.5 s), censored volumes and session means. Regressors were convolved with a canonical haemodynamic response function. The data and model were high-pass filtered at 1/128 Hz.

Two second-level whole-brain random-effect flexible factorial analyses were performed to look at sustained and transient patterns of activation. The first included the factors subject, age group and block type [(Baseline blocks − fixation blocks), (Reward blocks − fixation blocks)], modelling subject as a main effect and the age group x block type interaction. The second analysis similarly included the factors subject, age group and trial type (Baseline trials, No reward trials, Reward trials event-related activation).

Statistical contrasts were thresholded at *P* < 0.001 at the voxel-level with cluster size family-wise error (FWE) correction (*P* < 0.05) corresponding to a minimum cluster size of 82 voxels. In addition, activations that survived whole-brain FWE correction at *P* < 0.05 are indicated. Automatic anatomical labelling was done using AAL2 (Tzourio-Mazoyer *et al.,* 2002; Rolls *et al.,* 2015) and manual Brodmann area labelling with mricron (Rorden *et al.,* 2007). Regions that exhibited mixed sustained and transient effects were identified by running the transient contrasts inclusively masked by the sustained contrasts. Reversely, to identify regions that were exclusively sustained or transient, the relevant contrast was exclusively masked (*P*_uncorr_ < 0.05). Statistical maps for all whole-brain, voxel-wise analyses are available at https://neurovault.org/collections/4686/.

#### Regions of interest analyses

Region of interest (ROI) analyses were performed on extracted mean signal within regions that exhibited a mixed pattern of transient and sustained sensitivity to reward to explore possible interaction effects between task, condition and age group using the mixed block/event analysis parameter estimates. ROIs were defined using MarsBar (Brett *et al.,* 2002) as 10 mm radius spheres centred on the peak coordinates of clusters identified in the relevant contrasts.

## Results

### Behavioural results

**Fig. 2:**
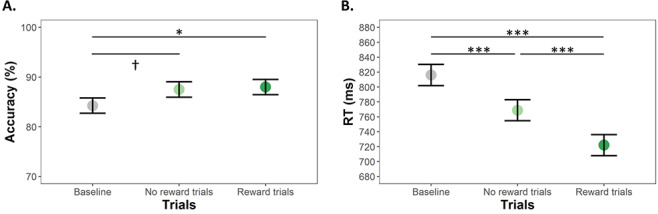
(Colour online) Mean accuracy (A) and RTs (B) as a function of trial type. Error bars represent Error bars represent SE. † < 0.10, ^*^P < 0.05, ^*^^*^P < 0.01, ^*^^*^^*^P < 0.001 (Tukey corrected).

Accuracy and speed in the letter-array WM task increased with age (Table 1) and differed between trial types (accuracy: *F*(1.7,84.3) = 4.20, *P* = 0.02, *η*_p_*^2^* = 0.02, RT: (*F*(1.6,77.9) = 40.50, *P* < 0.0001, *η*_p_*^2^* = 0.13). Participants were more accurate in Reward trials than in Baseline trials, with similar, but not significant, increased accuracy in No reward trials (Fig. 2A). Participants were faster in No reward trials than in Baseline trials and even faster for Reward trials (Fig. 2B).

**Table 1:** Summary statistics of measures collected in adolescent and adult participants

Measure	Adolescents (M ± SE)	Adults (M ± SE)	Age group comparisons
Letter-array WM task accuracy (%)	84.1 ± 1.7	90.3 ± 2.1	*F*(1,48) = 5.35, *P* = 0.03, *η*_p_*^2^* = 0.08
Letter-array WM task RT (ms)	795 ± 18	742 ± 18	*F*(1,48) = 3.98, *P* = 0.05, *η*_p_*^2^* = 0.06
Reward sensitivity composite^a^	0.21 ± 0.13	−0.19 ± 0.21	n.s. (*P* = 0.09)
Reward sensitivity SPSRQ (possible range: 0–16)^a^	8.4 ± 0.6	6.2 ± 0.7	*F*(1,48) = 4.4, *P* = 0.04 *η*_p_*^2^* = 0.09
Reward sensitivity BIS/BAS (possible range: 13–52)^a^	40.3 ± 1.3	39.9 ± 1.3	n.s. (*P* = 0.86)
Forward digit span total score (possible range: 1–22)	17.0 ± 0.6	18.1 ± 0.7	n.s. (*P* = 0.24)
Backward digit span total score (possible range: 1–22)	8.9 ± 0.6	11.8 ± 0.8	*F*(1,48) = 10.62, *P* < 0.002, *η*_p_*^2^* = 0.18
No go accuracy (%)	87.8 ± 1.7	91.7 ± 1.9	n.s. (*P* = 0.14)
WEBEXEC (possible range: 6–24)^b^	13.3 ± 0.5	12.9 ± 0.7	n.s. (*P* = 0.57)
Total money earned (£) (possible range: 0–8)	6.50 ± 0.24	6.67 ± 0.23	n.s. (*P* = 0.63)
Rating of monetary incentive (possible range: 1–5)^c^	4.17 ± 0.14	3.45 ± 0.30	*F*(1,48) *=* 6.02, *P* = 0.02, *η_p_*^2^ = 0.11

^a^Higher scores indicate more sensitivity to reward.

^b^Higher scores indicate more executive function failures.

^c^Higher scores indicate finding money more rewarding.

There was a trend for a decrease in reward sensitivity (z-score normalised composite index of the two self-report indices, SPSRQ and BIS/BAS) with age. A *post hoc* analysis revealed adolescents were more sensitive to rewards than adults when assessed with the SPSRQ, but not the BIS/BAS (Table 1). Adolescents and adults earned comparable amounts of money although adolescents reported finding money incentives more rewarding than adults (Table 1). Adults had greater backward digit span scores than adolescents, but the two age groups did not differ on the forward digit span task, WEBEXEC or Go/No go task (Table 1). When including backward digit span score as a covariate in the mixed design ANOVAs of the letter-array WM task, the difference between age groups in accuracy became non-significant (*F*(1,46) = 1.21, *P* = 0.28); however, the RT difference remained (*F*(1,46) = 5.82, *P* = 0.02).

### Neuroimaging results

#### Baseline activation during the WM task

**Fig. 3:**
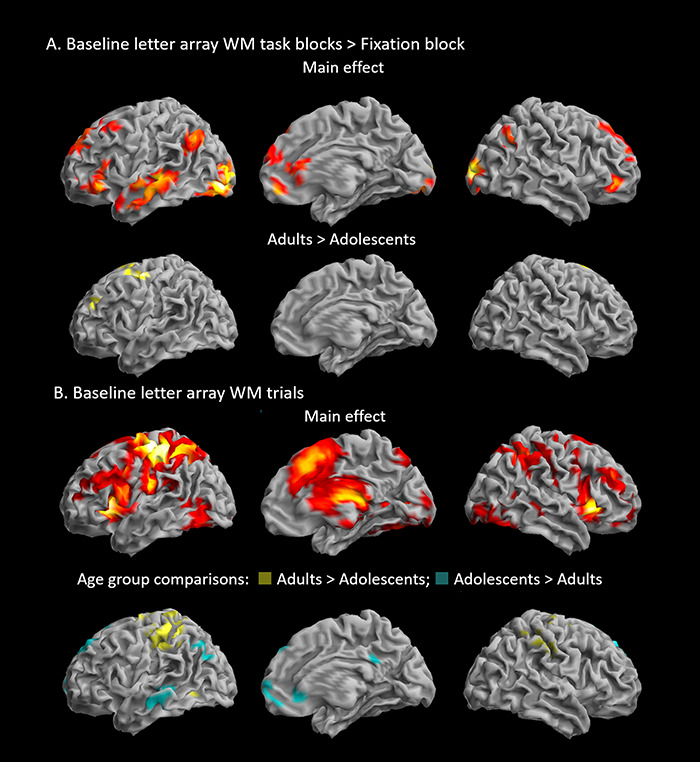
Letter working memory task activation and age group differences in the absence of reward. (A) Regions showing sustained increased BOLD signal in the Baseline task blocks *vs* fixation blocks. Top, main effect; bottom, interaction with age group. (B) Regions showing transient increased BOLD signal in Baseline trials. Top, main effect; bottom, interaction with age group. Contrasts are rendered on the surface of the SPM12 MNI template. Threshold: voxel *P*_uncorr_ = 0.001, cluster *P*_FWE_ < 0.05 (*k* = 82).

A broad network of regions showed *sustained* increased BOLD signal during letter-array WM task blocks compared to fixation blocks in the Baseline run (Table 2 and Fig. 3A). Activation was overall more extensive in the left hemisphere, which may reflect the verbal nature of the task but also overlapped with the ‘default mode network’. In the frontal lobes, bilateral activation was observed in the superior frontal gyri (SFG) and anterior part of the inferior frontal gyri (IFG), extending along the medial wall into the anterior aspect of the ACC. There was increased bilateral parietal activity in the left and right angular gyri, as well as in the left middle temporal gyri and medial and left inferior occipital gyri. Activation in the occipital cortex and angular gyrus bilaterally, left fusiform gyrus, middle temporal gyrus and temporal poles, inferior and superior frontal gyri and putamen survived voxel-level whole-brain correction (Table 2). Compared to adolescents, adults showed increased activation, which survived whole-brain correction at the cluster-level (but not voxel-level) in the left superior frontal and superior medial gyri, extending into the ACC, precentral gyrus and supplementary motor area (SMA), and activity in the lingual gyri (Table 2 and Fig. 3A).

**Table 2:** Letter-array working memory task neuroimaging results in the Baseline run

Region	L/R	Extent	*t*-score	*x*	*y*	*z*	BA
Baseline task blocks > Baseline fixation blocks							
Inferior occipital cortex	L	629^b^	10.09^a^	−27	−88	−10	18
Inferior occipital cortex	L		6.01^a^	−36	−67	−10	19
Fusiform gyrus	L		5.73^a^	−36	−46	−19	37
Mid occipital cortex	R	334^b^	8.63^a^	27	−97	5	17
Mid temporal gyrus	L	895^b^	6.90^a^	−60	−28	−1	21
Mid temporal gyrus	L		6.89^a^	−60	−10	−16	21
Mid temporal pole	L		4.33^a^	−48	11	−31	20
Mid superior frontal gyrus	L	2650^b^	6.82^a^	−9	50	50	9
Superior frontal gyrus	L		6.19^a^	−21	29	59	8
Inferior frontal gyrus	R		5.92^a^	12	32	5	11
Putamen	L	176^b^	5.97^a^	−21	2	5	
Angular gyrus	L	323^b^	5.64^a^	−42	−58	29	39
Angular gyrus	L		5.57^a^	−45	−67	47	39
Angular gyrus	R	125^b^	5.50^a^	51	−61	32	39
Adults [Baseline blocks − Baseline fixation blocks] > Adolescents [Baseline blocks − Baseline fixation blocks]							
Superior frontal gyrus	L	168^b^	4.64	−18	2	56	6
Precentral gyrus	L		4.58	−39	−4	56	6
SMA	L		3.49	−6	17	65	6
Medial superior frontal gyrus	L	102^b^	4.43	−9	17	41	32
ACC	L		3.76	−6	32	26	32
Lingual gyrus	L	160^b^	4.23	−18	−46	−7	30
Lingual gyrus	L		3.96	−21	−67	−13	18
Superior medial frontal gyrus	L	113^b^	4.21	−30	47	26	46
Superior frontal gyrus	L		4.05	−30	38	47	9
Baseline trials							
Precentral gyrus	L	18 304 ^b^	26.63^a^	−36	−7	68	6
Insula	L		24.92^a^	−30	20	5	48
Postcentral gyrus	L		23.83^a^	−45	−37	53	2
Middle frontal gyrus	R	530^b^	12.82^a^	42	38	32	46
Middle frontal gyrus	R		9.14^a^	39	56	14	46
Orbitofrontal cortex	R	97^b^	7.07^a^	21	44	−16	11
Adults Baseline trials > Adolescents Baseline trials							
Postcentral gyrus	L	695^b^	8.18^a^	−36	−43	62	2
Inferior parietal lobule	L		5.71^a^	−51	−28	50	2
Precuneus	L	339^b^	7.26^a^	−21	−46	8	37
Inferior temporal gyrus	L		5.80^a^	−39	−55	−7	37
Hippocampus	L		4.08	−36	−31	−10	37/20
Hippocampus	R	167^b^	6.23^a^	24	−40	8	37
Hippocampus	L		3.80	39	−37	−7	37
Superior frontal gyrus	R	364^b^	6.21^a^	33	−4	68	6
Paracentral lobule	R		6.09^a^	6	−19	80	6
Superior frontal gyrus	L		5.79^a^	−33	−4	68	6
Postcentral gyrus	R	148^b^	4.56	42	−31	50	3
Adolescents Baseline trials > Adults Baseline trials							
Medial orbitofrontal cortex		521^b^	7.4^a^	0	56	−4	10
Medial orbitofrontal cortex			6.70^a^	0	32	−10	11
Caudate			3.76	0	11	−13	25
Superior frontal gyrus	L	431^b^	7.05^a^	−9	47	47	9
Medial superior frontal gyrus			5.59^a^	0	44	20	32
Medial superior frontal gyrus	R		4.19	15	53	41	9
Mid temporal gyrus	L	250^b^	6.23^a^	−60	−25	−16	20
Posterior cingulate cortex	L	136^b^	4.60	−3	−55	32	23

Coordinates and *t*-values are listed for regions showing a significant difference in BOLD signal in the whole-brain analysis of block effects of WM [(Baseline blocks − Fixation blocks)] and trial effects of WM (Baseline trials). Both main effects and age group differences are reported. *x*, *y* and *z* = MNI coordinates. BA = Brodmann area, L/R = left/right hemisphere.

^a^Indicates voxels where *P*_FWE_ < 0.05 at the voxel-level.

^b^Indicates clusters where *P*_FWE_ < 0.05 at the cluster-level.

Widespread *transient* increased BOLD activation was observed in frontal, parietal and temporal regions, during WM task trials in the Baseline run, surviving both cluster-level and voxel-level whole-brain correction (Table 2 and Fig. 3B). In the frontal lobes, bilateral activation was observed in the SFG and anterior part of the IFG, as well as orbitofrontal cortex, extending along the medial wall predominantly into the middle cingulate cortex. There was increased bilateral activity in the insulae, in the angular gyri in the parietal cortex, as well as in the middle temporal gyri and inferior occipital gyri. Increases in subcortical activation were observed in the caudate and putamen, as well as in the thalamus and hippocampus bilaterally. There was widespread bilateral activation in the cerebellum. Adults showed increased activity in the precentral gyrus bilaterally, extending predominantly into the left postcentral gyrus compared to adolescents (left hemisphere peaks survived whole-brain correction at the voxel-level, except clusters in left hippocampus and postcentral gyrus), while adolescents exhibited less deactivation in medial PFC and precuneus than adults (Table 2 and Fig. 3B**)**.

#### Reward effects

**Fig. 4:**
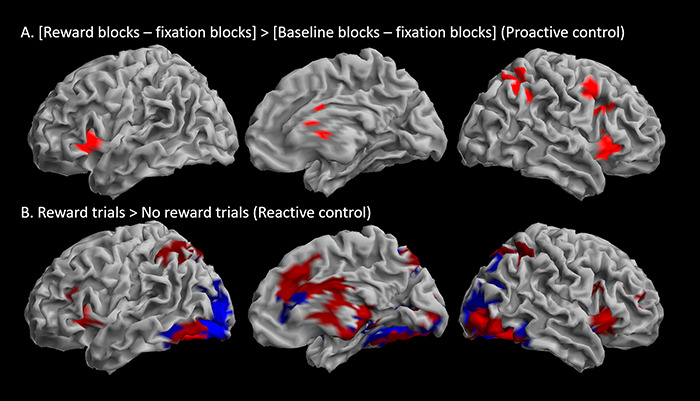
(Colour online) Sustained and transient effects of reward on letter-array working memory task activation. (A) Sustained effects identified by the [Reward blocks—fixation blocks] > [Baseline blocks—fixation blocks] contrast. (B) Transient effects identified by the Reward trials > No reward trials contrast. The red and blue shading of activations reflect whether these regions overall show transient activation or deactivations *vs* the implicit baseline. Contrasts are rendered on the surface of the SPM template. Threshold: voxel *P*_uncorr_ < 0.001, cluster *P*_FWE_ < 0.05 (*k* = 82).

*Sustained* effects of reward were assessed by contrasting task block activation of the Reward run and the Baseline run. This resulted in a large cluster peaking in the right insula and extending into bilateral ventral PFC, right dorsolateral PFC and the left insula, as well as into the caudate and putamen subcortically. There was an additional cluster in the right angular and supramarginal gyri. Bilateral ventroprefrontal, insula, caudate and occipital peaks survived whole-brain correction at the voxel-level (Table 3 and Fig. 4A). The pattern of activation largely did not overlap with the sustained WM task effects (Fig. 3A*vs*Fig. 4A). No increased activation was observed in the reverse contrast [Baseline blocks > Reward blocks].

*Transient* effects of reward were assessed by contrasting activation in Reward trials with No reward trials within the Reward run. This resulted in a large cluster peaking in inferior middle occipital gyrus and extending into the middle occipital gyrus, the superior and inferior parietal cortex bilaterally, right ventral and dorsolateral PFC and along the medial wall the medial frontal cortex and anterior and middle cingulate cortex, as well as the right insula. There were additional clusters in the left insula and right precentral and middle frontal gyri. Subcortical activity was observed bilaterally in the caudate nucleus extending slightly into accumbens, pallidum, thalamus, and bilateral hippocampi. There was widespread activation of cerebellar regions. Peaks located in posterior occipital brain regions, inferior parietal cortex, subcortical regions and the cerebellum survived whole-brain correction at the voxel-level. Among anterior brain regions, the ACC and insulae were the only peaks surviving voxel-level correction**.** No increased activation was observed in the reverse contrast [No reward trials > Reward trials] (Table 3 and Fig. 4B).

**Fig. 5:**
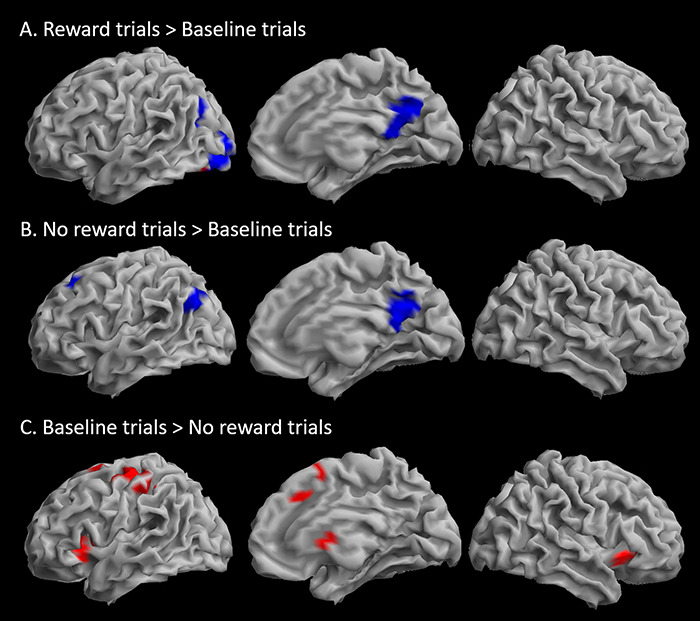
(Colour online) Differences in transient activation in Reward and No reward trials compared to Baseline trials. Increased transient activations in Reward (A) and No reward (B) trials compared to Baseline trials are observed mostly in regions showing overall deactivations compared to the implicit baseline. (C) Increased transient activation in Baseline trials compared to No reward trials was observed in regions showing overall activation *vs* the implicit baseline. The overall pattern shows intermediate activations for Baseline trials compared to No reward and Reward trials. Contrasts are rendered on the surface of the SPM template. Threshold: voxel *P*_uncorr_ < 0.001, cluster *P*_FWE_ < 0.05 (*k* = 82).

To further explore the pattern of changes in transient changes in BOLD signal according to reward, Reward and No reward trials were contrasted to Baseline trials (Table 3). Reward trials were associated with less deactivation of the precuneus and left lingual gyrus and middle occipital cortex than Baseline trials (the latter was not significant with voxel-level whole-brain correction) and greater activation than Baseline trials within a subset of the left lingual gyrus cluster (Fig. 5A). No difference in activation was observed in the reverse contrast [Baseline trials > Reward trials]. No reward trials showed, similarly to Reward trials, less deactivation in the precuneus than Baseline trials, as well as less deactivation than Baseline trials in the left superior frontal gyrus (the latter was not significant with voxel-level whole-brain correction) (Fig. 5B). Finally, Baseline trials showed higher activation than No reward trials in bilateral insulae, left precentral gyrus, medial frontal gyrus extending into middle cingulate gyrus and left inferior frontal cortex; there was also activation in the caudate and inferior and middle occipital gyri (Fig. 5C). Only activations in the bilateral insulae survived voxel-level whole-brain correction. The predominant pattern across regions showing transient increases in activation during the WM trials was therefore No reward trials < Baseline trials < Reward trials.

**Table 3:** Effects of reward on sustained and transient activation during the letter-array working memory task.

Region	L/R	Extent	*t*-score	*x*	*y*	*z*	BA
Proactive control: Reward block—fixation > Baseline run—fixation							
Insula	R	1152^b^	5.72^a^	30	20	−4	47
Insula	L		5.47^a^	−30	23	−4	47
Caudate	R		5.24^a^	9	8	14	
Insula	R		4.94	45	20	−7	38
Precentral gyrus	R		4.82	45	5	47	6
Pallidum	R		4.66	6	−1	2	
Middle frontal gyrus	R		4.31	48	20	29	44
Angular gyrus	R	356^b^	4.98	33	−61	47	7
Reactive control: Reward trials > No reward trials							
Inferior occipital cortex	L	11 426^b^	11.54^a^	−21	−91	−7	18
Lingual gyrus	L		11.48 ^a^	−24	−88	−10	18
Lingual gyrus	L		10.32^a^	−33	−82	−13	19
Middle occipital cortex	L		10.19 ^a^	−27	−91	2	18
Fusiform gyrus	L		9.47 ^a^	−33	−70	−13	19
Cerebellum	L		9.12 ^a^	−33	−58	−19	37
Vermis			7.69 ^a^	0	−64	−16	
Vermis	R		7.63 ^a^	3	−58	−28	
Inferior occipital cortex	R		7.34 ^a^	33	−88	−1	19
Cerebellum	R		7.18 ^a^	6	−64	−28	
Vermis			7.13 ^a^	0	−52	−13	
Vermis	R		6.89 ^a^	3	−43	−1	
ACC	R		7.36^a^	6	35	20	24
Insula	R		6.95^a^	36	20	−7	47
Insula	L	332^b^	6.42^a^	−30	23	2	47
Inferior frontal gyrus (triangular part)	L		4.00	−54	17	−4	38
Inferior frontal gyrus (triangular part)	R		3.53	−36	29	20	48
Precentral gyrus	R	97^b^	3.93	45	5	35	6
Frontal operculum	R		3.92	45	11	26	44
Frontal operculum	R		3.83	51	14	26	44
Inferior frontal gyrus (triangular part)	R		3.51	60	17	23	44
Middle frontal gyrus	R	94^b^	3.77	45	47	8	45
Middle frontal gyrus			3.69	39	56	17	46
Middle frontal gyrus			3.66	45	50	17	46
Middle frontal gyrus			3.64	45	38	23	45
Reward trials > Baseline trials							
Lingual gyrus	L	819^b^	7.42^a^	−18	−94	−7	18
Precuneus	L	854^b^	5.85^a^	−6	−58	29	23
Middle occipital cortex	L	107^b^	4.14	−36	−70	26	39
No reward trials > Baseline trials							
Precuneus	L	601^b^	6.83^a^	−6	−58	29	23
Middle occipital cortex	L	202^b^	5.35^a^	−48	−73	35	39
Superior frontal gyrus	L	94^b^	4.56	−21	38	50	9
Baseline trials > No reward trials							
Inferior frontal gyrus/insula	L	239^b^	5.76^a^	−39	20	−4	47
Inferior frontal gyrus/insula	R	120^b^	5.32^a^	36	23	−7	47
Precentral gyrus	L	346^b^	5.20^a^	−33	−10	62	6
Middle cingulate cortex	L	285^b^	5.15^a^	−9	20	38	32
SMA	L		4.97	−6	8	56	6
SMA	R		3.20	15	8	68	6
Caudate	R	182^b^	5.01^a^	3	5	2	
Inferior occipital gyrus	L	127^b^	4.73	−39	−67	−10	19
Middle occipital gyrus	R	202^b^	4.64	36	−85	−1	19
Fusiform gyrus	R		4.02	33	−61	−16	19
Mixed regions [(Reward block − fixation > Baseline run − fixation) inclusively masked by (Reward trials > No reward trials)							
Insula	R	225^b^	5.72^a^	30	20	−4	47
Insula	L	156^b^	5.47^a^	−30	23	−4	47
Angular gyrus	R	187^b^	4.98	33	−61	47	7
Caudate	R	109^b^	4.66	6	−1	2	
Exclusively transient regions [Reward trials > No reward trials] exclusively masked by [(Reward block—fixation) > (Baseline run—fixation)]							
Inferior occipital lobe	L	5371^b^	11.54^a^	−21	−91	−7	18
ACC	R	618^b^	6.79^a^	3	35	20	24
Inferior parietal lobule	L	112^b^	4.87	−45	−40	50	40
Insula	L	92^b^	4.82	42	11	−10	48

Coordinates and *t*-values are listed for regions showing a significant difference in BOLD signal for the whole-brain analysis for block effects of reward [(Reward run − Fixation) *vs* (Baseline run − Fixation)] and trial effect of reward [Reward trial *vs* No reward *vs* Baseline trials]. *x*, *y* and *z* = MNI coordinates. BA = Brodmann area, L/R = left/right hemisphere.

^a^Indicates voxels where *P*_FWE_ < 0.05 at the voxel-level.

^b^Indicates clusters where *P*_FWE_ < 0.05 at the cluster-level.

Inclusive and exclusive masking contrasts indicated that bilateral insulae (surviving voxel-wise whole-brain correction), right angular gyrus and a subcortical cluster including right caudate nucleus, thalamus and left pallidum exhibited reward context-related changes in both transient and sustained activity. No regions exhibited an exclusively sustained pattern of activation, but the more anterior aspect of the ACC as well as some cerebellar and occipital areas exhibited transient changes only in response to reward. Activations in the inferior occipital lobe and ACC survived voxel-level whole-brain correction (Table 3).

#### ROI analyses

**Fig. 6:**
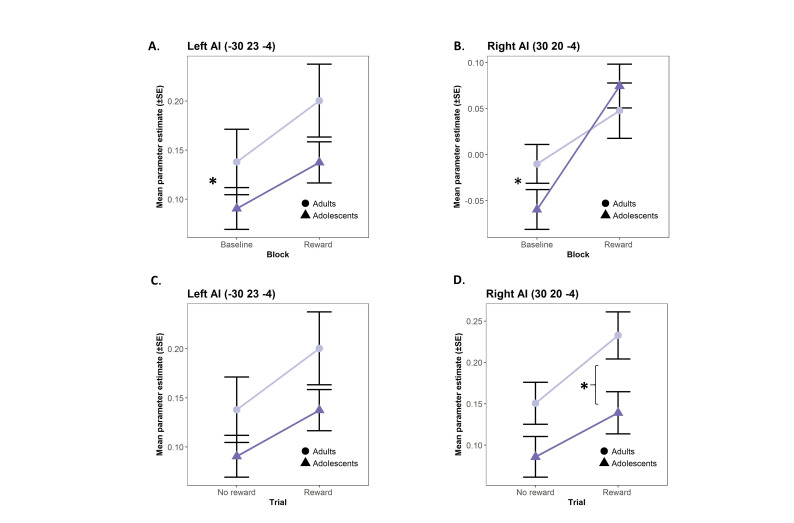
(Colour online) Reward effects on BOLD signal changes in the left and right AI. Parameter estimates extracted from 10 mm radius sphere regions of interests centred on the left and right AI are plotted to illustrate main effects of age and interactions between age and (A, B) block and (C, D) trial effects. Error bars represent SE. †*P* < 0.10, ^*^P < 0.05 (Tukey corrected).

To explore age effects in regions identified to have a mixed pattern of response to rewards, we extracted mean parameter estimates in 10 mm radius spheres centred on the peaks of the four clusters exhibiting modulation by reward of both transient and sustained activation (left and right insulae, angular gyrus and caudate, **see**Table 3). The left AI showed a significant interaction between run and age group: adolescents exhibited a greater increase in reward-dependent sustained activation than adults (*F*(1,48) = 6.35, *P* = 0.02, *η*_p_*^2^* = 0.05, Fig. 6A). The right AI showed a similar pattern (*F*(1,48) = 3.91, *P* = 0.05, *η*_p_*^2^* = 0.02) (Fig. 6B). Analyses of transient activations showed that adults exhibited increased overall activity in the right AI (*F*(1,48) = 4.79, *P* = 0.03, *η*_p_*^2^* = 0.08), but not the left AI (*F*(1,48) = 2.22, *P* = 0.14, *η*_p_*^2^* = 0.04), across Reward and No reward trials compared to adolescents (Fig. 6C and D). No other age effects were identified.

## Discussion

We examined the impact of reward on sustained and transient engagement of cognitive control, and whether differences exist between adolescence and adulthood. In the letter-array WM task, high accuracy rates can be achieved with a reactive control strategy. However, to produce accurate responses that are *fast enough*, the optimal strategy is to proactively sustain the task set and rule use across trials, in anticipation of the stimuli (Jimura *et al.,* 2010). Results showed similar behavioural and neural evidence for engagement of proactive and reactive strategies in the context of reward for adolescents and adults.

### Proactive control

RTs were faster in No reward than Baseline trials, suggesting sustained performance improvement in the Reward run associated with a proactive cognitive control strategy (Jimura *et al.,* 2010). Reward blocks were associated with increased frontoparietal activity outside of the network recruited in the main WM block contrast. Our results align with findings of reward-related increased sustained activity in the right lateral PFC (middle frontal gyrus) and right PPC (angular gyrus), regions associated with proactive control in adults (Locke and Braver, 2008; Jimura *et al.,* 2010) and children, adolescents and adults (Strang and Pollak, 2014). An increased cognitive control system engagement could also be reflective of higher load due to the introduction of two different reward conditions, Reward and No reward trials. However, this is unlikely to reflect a *task-switching* load, as the WM task stays constant [there is no perceptual, response or set shifting needed (Kim *et al.,* 2012)], and we see a pattern of improved performance rather than the performance cost typically associated with a switching context (Monsell, 2003). In addition, there was evidence for sustained activation in regions typically associated with reward across age groups: the caudate nucleus, putamen and orbitofrontal cortex (Silverman *et al.,* 2015).

### Reactive control

Adolescents and adults were fastest for Reward trials, with a similar trend for accuracy, which points to a trial-by-trial reward enhancement reflecting reactive control. Reward trials were associated with increased transient activity in cortical regions recruited overall during the letter-array WM task (AI, ACC and parietal cortex), in contrast to the block effects of reward, which did not overlap with WM regions. The overall pattern shows intermediate activations for Baseline trials compared to No reward and Reward trials.

A possible interpretation of these results is that, as transient activation is not as resource consuming as sustained activation, there is still scope for increased trial-by-trial recruitment of WM regions to increase the chance of obtaining a reward, on top of WM transient activation. In addition to the sustained activation, Reward trials also recruited the right orbitofrontal cortex, the caudate nucleus and putamen (Haber and Knutson, 2010).

In the present study, transient activity in the ACC might be related to increased monitoring in response to reward. The ACC has been proposed to be involved in performance monitoring and, when conflict arises, is thought to recruit higher control order structures in the lateral PFC (Botvinick and Braver, 2015). In this case, conflict might signal a cost–benefit analysis where cost of task performance is weighed against expect values of its outcome and necessary effort required (Shenhav *et al.,* 2013). Further, exclusively transient activations were observed in anterior aspects of the ACC, which fit with previous evidence that this region is selectively involved in compensatory reactive control processes (Jiang *et al.,* 2015).

### Mixed and exclusively transient regions

Bilateral insulae, the right PPC and the caudate exhibited a mixed pattern: with both higher sustained activity for the Reward run than the Baseline run and higher transient increases in activity for Reward trials compared to No reward trials, with Baseline trials showing intermediary levels of transient activity (i.e. No reward < Baseline < Reward in most cases). The AI emerged as the key mixed region involved in proactive and reactive control in response to reward, while the DLPFC had a more sustained pattern of activation in the baseline WM run. Sustained activation in the DLPFC in the reward context may reflect increased top-down ‘boosting’ excitatory connectivity to more posterior regions, which may facilitate maintenance of representations over a delay (Edin *et al.,* 2009). The AI has been implicated in top-down control processes including task-set maintenance (Dosenbach *et al.,* 2008; Nelson *et al.,* 2010) and tracking cognitive control demand stability (Jiang *et al.,* 2015). It has also been implicated in bottom-up salience detection of relevant cues (Menon and Uddin, 2010) as part of the salience network and key cognitive–emotional hub (Menon and Uddin, 2010; Smith *et al.,* 2014). It has been suggested that the AI may support the transient detection of salient stimuli and initiating attentional control signals which are then sustained by the ACC and the ventrolateral and DLPFC (Menon and Uddin, 2010). As one of the most commonly activated areas in fMRI studies, the AI has been implicated in controlling attention as a function of task demands (see Nelson *et al.,* 2010 for a review).

The caudate nucleus has been implicated in processing extrinsic reward related to monetary gains and losses (Haber and Knutson, 2010; Richards *et al.,* 2013). We found that activity in the caudate nucleus was greater in Reward blocks than Baseline blocks and that it tracked trial reward status as baseline activity was maintained for Reward trials only, while activation was lower for No reward trials. A speculative interpretation of these results is that once the explicit reward trials were introduced, the value of the No reward trials dropped compared to their starting level. However, the caudate nucleus has also been found to be activated in WM tasks in the absence of rewards (e.g. Ziermans *et al.,* 2012), and in the present study there were transient increases in activation in the caudate in Baseline WM task trials, which suggests a non-exclusive reward role of the caudate.

Our results speak to the debate surrounding two underlying configurations which have been proposed for the DMC (Jiang *et al.,* 2015). One of the accounts proposes that proactive and reactive control are implemented by different dynamics within the same region of the right dorsolateral PFC (BA 46/9) (Braver *et al.,* 2009; Burgess and Braver, 2010; Jimura *et al.,* 2010). Other accounts propose that different strategies are implemented by distinct brain regions (De Pisapia and Braver, 2006; Jiang *et al.,* 2015). Here, we found evidence that both mechanisms might be at play. The bilateral AI, right PPC and caudate nucleus showed a mixed pattern of response while the anterior aspects of the ACC showed transient effects of reward only.

### Developmental effects

In line with developmental studies of cognitive control (Luna *et al.,* 2015; Humphrey and Dumontheil, 2016), adults had greater overall accuracy and faster RT than adolescents, as well as greater backward digit span scores (Karakas *et al.,* 2002). However, since speed thresholds were determined individually, adolescents and adults earned comparable monetary rewards. Adolescents reported finding monetary incentives more rewarding than adults. Comparable performance between adolescents and adults might be driven by increased motivation to perform by the adolescents, perhaps associated with finding money more rewarding.

Adolescents showed a trend for more reward sensitivity than adults (Galván, 2013; van Duijvenvoorde *et al.,* 2016). *Post hoc* analyses revealed adolescents had significantly greater reward sensitivity than adults on the SPSRQ (Torrubia *et al.,* 2001, which assesses reward sensitivity *per se*, but not on the approach motivation subscale of the BIS/BAS (Carver and White, 1994). However, we did not find age differences in reward-related brain activation. Although age differences between adolescents and adults are often described in the neuroimaging reward literature, they are not consistent across stages of reward processing or type of task (Galván, 2010, 2013).

In the absence of rewards, previous developmental work has suggested that adolescents employ a more reactive than proactive cognitive control strategy (Velanova *et al.,* 2009; Andrews-Hanna *et al.,* 2011; Alahyane *et al.,* 2014). In the present study, we show that, like Strang and Pollack (2014), in the context of potential rewards, adolescents, like adults, can sustain cognitive control proactively. By also examining reactive control, in contrast to Strang and Pollack (2014), we provide evidence that adolescents, like adults, show additional improvements in a trial-by-trial fashion.

In follow-up ROI analyses of the main results, we found that transient activation was overall greater in the right AI in adults than in adolescents but that both age groups showed similar increases in transient activation in Reward trials compared to No reward trials. A different pattern was observed for sustained activation, whereby adolescents showed a greater increase in activation in task blocks of the Reward run compared to the Baseline run in the left AI, ‘catching up’ with adult levels of activation. Adolescents may be relying on an adaptive mechanism of sustained, but not transient, increase in AI activation. This speaks to an immature proactive capacity in adolescents that is only engaged in the context of reward. The role of the AI in adolescent decision-making processes is increasingly recognised, suggesting that the relative immaturity of this cognitive–emotional hub, which is connected to both the lateral PFC and striatum, may bias adolescents in affectively driven contexts (for a review, see Smith *et al.,* 2014). Here we suggest that a sensitivity to reward context in the AI may support increased sustained engagement of cognitive control in some instances.

### Limitations and future directions

Varying between Reward and No reward trials could be an additional component of the task which may have led to an increase in sustained activation of the cognitive control system by making the task more engaging. However, a greater overall engagement could not account for transient differences between Reward and No reward trials. Order effects are a limitation of the current study (as in Jimura *et al.,* 2010). Counterbalancing the order of blocks was not possible to ensure participants were at first naïve regarding potential rewards to determine their baseline performance. To minimise order effects related to practice, we introduced a long practice period to ensure that participant’s performance stabilised before the scanning runs. It is possible that the better performance observed in the second run could still be driven in part by practice effects. Plots of RT and accuracy as a function of trial number and block number suggest indeed that in adolescents the RT difference between Baseline and No reward trials may has been driven by practice effects (Supplementary Figure S1). However, this is not apparent in adults, nor in the accuracy data (Supplementary Figure S2). Practice effects however could not explain the difference in RT and brain activation between Reward and No reward trials. Although demanding, the task was not very difficult, as reflected by high accuracy rates. It might be that the balance between proactive and reactive strategies begins to emerge in more challenging cognitive control tasks, and future studies could investigate this.

## Conclusion

This study shows behavioural and neuroimaging evidence of modulation of both proactive and reactive control by reward in adults and in adolescents. Proactive and reactive control were found to be supported both by partly separable frontoparietal neural circuitries and by regions that exhibit both sustained and transient modulation by reward. In the face of incentives, adolescents and adults can sustain cognitive control in a proactive fashion, with additional transient readjustments in response to the reward. There is some evidence of adaptive higher sustained activation in the AI by adolescents in the context of reward.

## Conflict of interest

The authors have no conflict of interest to declare.

## Funding

This work was supported by the Mexican National Council for Science and Technology (CONACYT) [465015 to L.M.W].

## Supplementary Material

File_nsz093

## References

[ref1] Alahyane N., Brien D.C., Coe B.C., Stroman P.W., Munoz D.P. (2014). Developmental improvements in voluntary control of behavior: effect of preparation in the fronto-parietal network? NeuroImage, 98, 103–17. doi: 10.1016/j.neuroimage.2014.03.008. 24642280

[ref2] Andrews-Hanna J.R., Seghete K.L., Claus E.D., Burgess G.C., Ruzic L., Banich M.T. (2011). Cognitive control in adolescence: neural underpinnings and relation to self-report behaviors. PLoS One, 6(6), 27–30. doi: 10.1371/journal.pone.0021598. PMC3125248 21738725

[ref3] Blackwell K.A., Munakata Y. (2014). Costs and benefits linked to developments in cognitive control. Developmental Science, 17(2), 203–11. doi: 10.1111/desc.12113. 24329774 PMC4078978

[ref4] Botvinick M.M., Braver T.S. (2015). Motivation and cognitive control: from behavior to neural mechanism. Annual Review of Psychology, 66(1), 83–113. doi: 10.1146/annurev-psych-010814-015044. 25251491

[ref5] Brahmbhatt S.B., White D.A., Barch D.M. (2010). Developmental differences in sustained and transient activity underlying working memory. Brain Research, 1354, 140–51. doi: 10.1016/j.brainres.2010.07.055. 20659432 PMC2940123

[ref6] Braver T.S. (2012). The variable nature of cognitive control: a dual mechanisms framework. Trends in Cognitive Sciences, 16(2), 106–13. doi: 10.1016/j.tics.2011.12.010. 22245618 PMC3289517

[ref7] Braver T.S., Paxton J.L., Locke H.S., Barch D.M. (2009). Flexible neural mechanisms of cognitive control within human prefrontal cortex. Proceedings of the National Academy of Sciences of the United States of America, 106(18), 7351–6. doi: 10.1073/pnas.0808187106. 19380750 PMC2678630

[ref8] Braver T.S., Krug M.K., Chiew K.S., et al. (2014). Mechanisms of motivation–cognition interaction: challenges and opportunities. Cognitive, Affective, & Behavioral Neuroscience, 14(2), 443–72. doi: 10.3758/s13415-014-0300-0. PMC4986920 24920442

[ref9] Brett M., Anton J.-L., Valabregue R., Poline J.-B. (2002). Region of interest analysis using an SPM toolbox. Presented at the 8th International Conference on Functional Mapping of the Human Brain, 2–6 June 2002, Sendai, Japan*.*

[ref10] Buchanan T., Heffernan T.M., Parrott A.C., Ling J., Rodgers J., Scholey A.B. (2010). A short self-report measure of problems with executive function suitable for administration via the internet. Behavior Research Methods, 42(3), 709–14. doi: 10.3758/BRM.42.3.709. 20805593

[ref11] Burgess G.C., Braver T.S. (2010). Neural mechanisms of interference control in working memory: effects of interference expectancy and fluid intelligence. PLoS One, 5(9), e12861. doi: 10.1371/journal.pone.0012861. 20877464 PMC2942897

[ref12] Carver C.S., White T.L. (1994). Behavioural inhibition, behavioural activation, and affective responses to impending reward and punishment: the BIS/BAS scales. Journal of Personality and Social Psychology, 67, 319–33.

[ref13] Casey B.J. (2015). Beyond simple models of self-control to circuit-based accounts of adolescent behavior. Annual Review of Psychology, 66(1), 295–319. doi: 10.1146/annurev-psych-010814-015156. 25089362

[ref14] Cauley S.F., Polimeni J.R., Bhat H., Wald L.L., Setsompop K. (2014). Interslice leakage artifact reduction technique for simultaneous multislice acquisitions. Magnetic Resonance in Medicine, 72(1), 93–102. doi: 10.1002/mrm.24898. 23963964 PMC4364522

[ref15] Chatham C.H., Frank M.J., Munakata Y. (2009). Pupillometric and behavioral markers of a developmental shift in the temporal dynamics of cognitive control. Proceedings of the National Academy of Sciences, 106(14), 5529–33. doi: 10.1073/pnas.0810002106. PMC2666994 19321427

[ref16] Chevalier N., Martis S.B., Curran T., Munakata Y. (2015). Metacognitive processes in executive control development: the case of reactive and proactive control. Journal of Cognitive Neuroscience, 27(6), 1125–36. doi: 10.1162/jocn_a_00782. 25603026 PMC4510990

[ref17] Chevalier N., Jackson J., Revueltas Roux A., Moriguchi Y., Auyeung B. (2019). Differentiation in prefrontal cortex recruitment during childhood: evidence from cognitive control demands and social contexts. Developmental Cognitive Neuroscience, 36, 100629. doi: 10.1016/j.dcn.2019.100629. 30913498 PMC6969260

[ref18] Chiew K.S., Braver T.S. (2013). Temporal dynamics of motivation-cognitive control interactions revealed by high-resolution pupillometry. Frontiers in Psychology, 4(1), 15. doi: https://doi.org/10.3389/fpsyg.2013.00015. 23372557 10.3389/fpsyg.2013.00015 PMC3557699

[ref19] Chiew K.S., Braver T.S. (2017). Context processing and control in the human brain: from gating models to dual mechanisms In: Egner T., editor. The Wiley Handbook of Cognitive Control, Hoboken: Wiley Blackwell, pp. 143–66.

[ref20] Cohen A.O., Breiner K., Steinberg L., et al. (2016). When is an adolescent an adult? Assessing cognitive control in emotional and nonemotional contexts. Psychological Science, 27(4), 549–62. doi: 10.1177/0956797615627625. 26911914

[ref21] Crone E.A., Dahl R.E. (2012). Understanding adolescence as a period of social–affective engagement and goal flexibility. Nature Reviews Neuroscience, 13(9), 636–50. doi: 10.1038/nrn3313. 22903221

[ref22] De Pisapia N., Braver T.S. (2006). A model of dual control mechanisms through anterior cingulate and prefrontal cortex interactions. Neurocomputing, 69, 1322–6.

[ref23] Dosenbach N.U.F., Fair D.A., Cohen A.L., Schlaggar B.L., Petersen S.E. (2008). A dual-networks architecture of top-down control. Trends in Cognitive Sciences, 12(3), 99–105. doi: 10.1016/j.tics.2008.01.001. 18262825 PMC3632449

[ref24] van Duijvenvoorde A.C.K., Peters S., Braams B.R., Crone E.A. (2016). What motivates adolescents? Neural responses to rewards and their influence on adolescents’ risk taking, learning, and cognitive control. Neuroscience & Biobehavioral Reviews, 70, 135–47. doi: 10.1016/j.neubiorev.2016.06.037. 27353570

[ref25] Edin F., Klingberg T., Johansson P., McNab F., Tegnér J., Compte A. (2009). Mechanism for top-down control of working memory capacity. Proceedings of the National Academy of Sciences, 106(16), 6802–7. doi: 10.1073/pnas.0901894106. PMC2672558 19339493

[ref26] Galván A. (2010). Adolescent development of the reward system. Frontiers in Neuroscience, 4, 6–14. doi: 10.3389/neuro.09.006.2010. 20179786 PMC2826184

[ref27] Galván A. (2013). The teenage brain: sensitivity to rewards. Current Directions in Psychological Science, 22(2), 88–93. doi: 10.1177/0963721413480859. PMC3992953 24761055

[ref28] Geier C., Terwilliger R., Teslovich T., Velanova K., Luna B. (2010). Immaturities in reward processing and its influence on inhibitory control in adolescence. Cerebral Cortex, 20(7), 1613–29. doi: 10.1093/cercor/bhp225. 19875675 PMC2882823

[ref29] Haber S.N., Knutson B. (2010). The reward circuit: linking primate anatomy and human imaging. Neuropsychopharmacology, 35(1), 4–26. doi: 10.1038/npp.2009.129. 19812543 PMC3055449

[ref30] Humphrey G., Dumontheil I. (2016). Development of risk-taking, perspective-taking, and inhibitory control during adolescence. Developmental Neuropsychology, 41(1–2), 59–76. doi: 10.1080/87565641.2016.1161764. 27070826

[ref31] Insel C., Kastman E.K., Glenn C.R., Somerville L.H. (2017). Development of corticostriatal connectivity constrains goal-directed behavior during adolescence. Nature Communications, 8(1), 1605. doi: 10.1038/s41467-017-01369-8. PMC5705718 29184096

[ref32] Jiang J., Beck J., Heller K., Egner T. (2015). An insula-frontostriatal network mediates flexible cognitive control by adaptively predicting changing control demands. Nature Communications, 6(5), 8165. doi: 10.1038/ncomms9165. PMC4595591 26391305

[ref33] Jimura K., Locke H.S., Braver T.S. (2010). Prefrontal cortex mediation of cognitive enhancement in rewarding motivational contexts. Proceedings of the National Academy of Sciences of the United States of America, 107(19), 8871–6. doi: 10.1073/pnas.1002007107. 20421489 PMC2889311

[ref34] Karakas S., Yalin A., Irak M., Erzengin Ö.U. (2002). Digit span changes from puberty to old age under different levels of education. Developmental Neuropsychology, 22(2), 423–53. doi: 10.1207/S15326942DN2202_1. 12537332

[ref35] Kim C., Cilles S.E., Johnson N.F., Gold B.T. (2012). Domain general and domain preferential brain regions associated with different types of task switching: a meta-analysis. Human Brain Mapping, 33(1), 130–42. doi: 10.1002/hbm.21199. 21391260 PMC3421461

[ref36] Krebs R.M., Hopf J.-M., Boehler C.N. (2015). Within-trial effects of stimulus-reward associations In: Braver T.S., editor. Motivation and Cognitive Control, 1st edn, Routledge: Taylor & Francis Group.

[ref37] Locke H.S., Braver T.S. (2008). Motivational influences on cognitive control: behavior, brain activation, and individual differences. Cognitive, Affective, & Behavioral Neuroscience, 8(1), 99–112. doi: 10.3758/CABN.8.1.99. 18405050

[ref38] Luna B., Marek S., Larsen B., Tervo-Clemmens B., Chahal R. (2015). An integrative model of the maturation of cognitive control. Annual Review of Neuroscience, 38(1), 151–70. doi: 10.1146/annurev-neuro-071714-034054. PMC5661874 26154978

[ref39] Marklund P., Fransson P., Cabeza R., Petersson K.M.K., Ingvar M., Nyberg L. (2007). Sustained and transient neural modulations in prefrontal cortex related to declarative long-term memory, working memory, and attention. Cortex, 43(1), 22–37. doi: 10.1016/S0010-9452(08)70443-X. 17334205

[ref40] McDaniel M.A., LaMontagne P., Beck S.M., Scullin M.K., Braver T.S. (2013). Dissociable neural routes to successful prospective memory. Psychological Science, 24(9), 1791–800. doi: 10.1177/0956797613481233. 23907544 PMC4398313

[ref41] Menon V., Uddin L.Q. (2010). Saliency, switching, attention and control: a network model of insula function. Brain Structure & Function, 214(5–6), 655–67. doi: 10.1007/s00429-010-0262-0. 20512370 PMC2899886

[ref42] Monsell S. (2003). Task switching. Trends in Cognitive Sciences, 7(3), 134–40. doi: 10.1016/S1364-6613(03)00028-7. 12639695

[ref43] Nelson S.M., Dosenbach N.U.F., Cohen A.L., Wheeler M.E., Schlaggar B.L., Petersen S.E. (2010). Role of the anterior insula in task-level control and focal attention. Brain Structure and Function, 214(5–6), 669–80. doi: 10.1007/s00429-010-0260-2. 20512372 PMC2886908

[ref44] Padmanabhan A., Geier C., Ordaz S.J., Teslovich T., Luna B. (2011). Developmental changes in brain function underlying the influence of reward processing on inhibitory control. Developmental Cognitive Neuroscience, 1(4), 517–29. doi: 10.1016/j.dcn.2011.06.004. 21966352 PMC3181104

[ref71] R Core Team (2018). R: A language and environment for statistical computing. R Foundation for Statistical Computing, Vienna, Austria Retrieved from https://CRAN.R-project.org/package=afex.

[ref45] Richards J.M., Plate R.C., Ernst M. (2013). A systematic review of fMRI reward paradigms used in studies of adolescents vs. adults: the impact of task design and implications for understanding neurodevelopment. Neuroscience and Biobehavioral Reviews, 37(5), 976–91. doi: 10.1016/j.neubiorev.2013.03.004. 23518270 PMC3809756

[ref46] Rolls E.T., Joliot M., Tzourio-Mazoyer N. (2015). Implementation of a new parcellation of the orbitofrontal cortex in the automated anatomical labeling atlas. NeuroImage, 122, 1–5. doi: 10.1016/J.NEUROIMAGE.2015.07.075. 26241684

[ref47] Rorden C., Karnath H.-O., Bonilha L. (2007). Improving lesion-symptom mapping. Journal of Cognitive Neuroscience, 19, 1081–8. 17583985 10.1162/jocn.2007.19.7.1081

[ref72] Siegel J.S., Power J.D., Dubis J.W., et al (2014). Statistical improvements in functional magnetic resonance imaging analyses produced by censoring high-motion data points. Hum. Brain Mapp, 35, 1981–96. doi: 10.1002/hbm.22307. 23861343 PMC3895106

[ref48] Shenhav A., Botvinick M.M., Cohen J.D. (2013). The expected value of control: an integrative theory of anterior cingulate cortex function. Neuron, 79(2), 217–40. doi: 10.1016/j.neuron.2013.07.007. 23889930 PMC3767969

[ref49] Silverman M.H., Jedd K., Luciana M. (2015). Neural networks involved in adolescent reward processing: an activation likelihood estimation meta-analysis of functional neuroimaging studies. NeuroImage, 122, 427–39. doi: 10.1016/j.neuroimage.2015.07.083. 26254587 PMC4618189

[ref50] Simmonds D.J., Pekar J.J., Mostofsky S.H. (2008). Meta-analysis of go/no-go tasks demonstrating that fMRI activation associated with response inhibition is task-dependent. Neuropsychologia, 46(1), 224–32. doi: 10.1016/j.neuropsychologia.2007.07.015. 17850833 PMC2327217

[ref51] Singmann H., Bolker B., Westfall J., Aust F. (2018). Afex: analysis of factorial experiments*.* [R package version 0.22-1] Retrieved from https://CRAN.R-project.org/package=afex

[ref52] Smith A.R., Steinberg L., Chein J.M. (2014). The role of the anterior insula in adolescent decision making. Developmental Neuroscience, 36(3–4), 196–209. doi: 10.1159/000358918. 24853135 PMC5544351

[ref53] Strang N.M., Pollak S.D. (2014). Developmental continuity in reward-related enhancement of cognitive control. Developmental Cognitive Neuroscience, 10, 34–43. doi: 10.1016/j.dcn.2014.07.005. 25160678 PMC4332542

[ref54] Torrubia R., Ávila C., Moltó J., Caseras X. (2001). The sensitivity to punishment and sensitivity to reward questionnaire (SPSRQ) as a measure of Gray’s anxiety and impulsivity dimensions. Personality and Individual Differences, 31(6), 837–62. doi: 10.1016/S0191-8869(00)00183-5.

[ref55] Tzourio-Mazoyer N., Landeau B., Papathanassiou D., et al. (2002). Automated anatomical labeling of activations in SPM using a macroscopic anatomical parcellation of the MNI MRI single-subject brain. NeuroImage, 15(1), 273–89. doi: 10.1006/nimg.2001.0978. 11771995

[ref56] Velanova K., Wheeler M.E., Luna B. (2009). The maturation of task set-related activation supports late developmental improvements in inhibitory control. Journal of Neuroscience, 29(40), 12558–67. doi: 10.1523/JNEUROSCI.1579-09.2009. 19812330 PMC2788337

[ref57] Visscher K.M., Miezin F.M., Kelly J.E., et al. (2003). Mixed blocked/event-related designs separate transient and sustained activity in fMRI. NeuroImage, 19(4), 1694–708. 12948724 10.1016/s1053-8119(03)00178-2

[ref58] Wechsler D. (2011). Weschler Abbreviated Scale of Intelligence (WASI-II), San Antonio: NCS Pearson. doi. 10.1177/0734282912467756.

[ref59] Xu J., Moeller S., Auerbach E.J., et al. (2013). Evaluation of slice accelerations using multiband echo planar imaging at 3 T. NeuroImage, 83, 991–1001. doi: 10.1016/j.neuroimage.2013.07.055. 23899722 PMC3815955

[ref60] Zanolie K., Crone E.A. (2018). Development of Cognitive Control across Childhood and Adolescence In: Ghetti S., editor. The Stevens’ Handbook of Experimental Psychology and Cognitive Neuroscience, 4th edn, Vol. 4, New York: Wiley.

[ref61] Zhai Z.W., Pajtek S., Luna B., Geier C., Ridenour T.A., Clark D.B. (2015). Reward-modulated response inhibition, cognitive shifting, and the orbital frontal cortex in early adolescence. Journal of Research on Adolescence, 25(4), 753–64. doi: 10.1111/jora.12168. 26755891 PMC4705559

[ref62] Ziermans T., Dumontheil I., Roggeman C., et al. (2012). Working memory brain activity and capacity link MAOA polymorphism to aggressive behavior during development. Translational Psychiatry, 2(2), e85. doi: 10.1038/tp.2012.7. 22832821 PMC3309555

