# Supplementary material for: Rewards Enhance Proactive and Reactive Control in Adolescence and Adulthood
Source: Soc Cogn Affect Neurosci. 2019 Dec 10;14(11):1219–32. doi: 10.1093/scan/nsz093 (PMC7057287; doi:10.1093/scan/nsz093)
Supplement: File_nsz093 [file file_nsz093.docx]

# **Supplementary information**

# Magis-Weinberg et al. Rewards enhance proactive and reactive control in adolescence and adulthood

## **SI Methods**

### **Experimental Design and Stimulus Material**

### **Procedure**

#### **Letter array working memory task**

We employed a fixed set-size Sternberg-item recognition task that was adapted from Jimura, Locke and Braver (2010; **Figure 1B**). At the beginning of each working memory trial, a cue was presented in the centre of the screen for 1.5 s. There were two different cues: **“⋆⋆⋆ trial**” indicating that a potential reward could be obtained on this trial (*Reward trial*) or “**new trial**” indicating that no reward could be obtained on this trial (*Baseline trial* or *No reward trial*). As in Jimura et al. (2010), immediately after the reward cue, without jittering, five uppercase consonants were presented on the screen for a 1.5 s encoding period. A fixed 3.5 s delay followed to serve as a retention interval. After the delay, a single lowercase probe letter was presented for a fixed duration of 1.5 s. Participants were required to indicate whether the probe matched one of the letters from the memory set. Participants were encouraged to respond both accurately and quickly. Responses were indicated by pressing one of two buttons on a handheld response box (right index finger = match, right middle finger = no match), and were followed by a fixed 2 s delay and then feedback for a fixed duration of 1.5 s. Thus, total trial duration was 11.5 s (excluding intertrial interval). Four different types of feedback could be provided, indicating whether the response was incorrect, too slow, correct and not rewarded, or correct and rewarded (**Figure 1B**). Cut-off times were individually set for each participant, based on their own median correct reaction time (RT) on trials performed in the practice (see Procedure). Trials were separated by intertrial intervals lasting 2.5, 5 or 7.5 s.

### ***MRI data preprocessing***

Realignment estimates were used to calculate framewise displacement (FD) for each volume, which is a composite, scalar measure of head motion across the six realignment estimates (Siegel et al., 2014). Volumes with an FD > 0.9 mm were censored and excluded from general linear model (GLM) estimation by including a regressor of no interest for each censored volume. No session met criteria for exclusion (scanning sessions with more than 10 % of volumes censored or a root mean square (RMS) movement over the whole session greater than 1.5 mm). Adolescent and adult participants did not differ significantly in the number of overall censored volumes (adolescents = 0.75 ± 1.21 (SD), adults = 0.56 ± 1.87; *p* = .659), mean RMS rotational movement (adolescents = 0.18 mm ± 0.07, adults = 0.19 mm ± 0.08; *p* = .633), and mean FD (adolescents = 0.11 mm ± 0.03, adults = 0.10 mm ± 0.05; *p* = .280). There was a difference between groups in terms of mean RMS translational movement, with more movements in this axis for adults than adolescents (adolescents = 0.18 mm ± 0.07, adults = 0.24 mm ± 0.12; *p* = .025).

## **SI Results**

To investigate whether some of the differences in performance between Baseline, No reward and Reward trials may have been due to practice effects of the course of the two scanning runs, which were performed in a fixed order (Baseline, then Reward run), mean reaction time and accuracy were plotted as a function of trial number over the course of the difference practice and test phases of the working memory task.

While practice effects are clearly observed in both accuracy and RT during the second, time-limited, practice phase (**Figures S1** and **S2**). Practice effects are subtler during the test (scanning runs) but do suggest that in adolescents in particular faster RTs in No reward trials compared to Baseline trials may be due to practice effects. This is not apparent in the adult group and the accuracy data does not demonstrate consistent improvements in performance over the course of successive trials of the Baseline and Reward runs. Note that formal statistical analyses were not performed on the data plotted here.


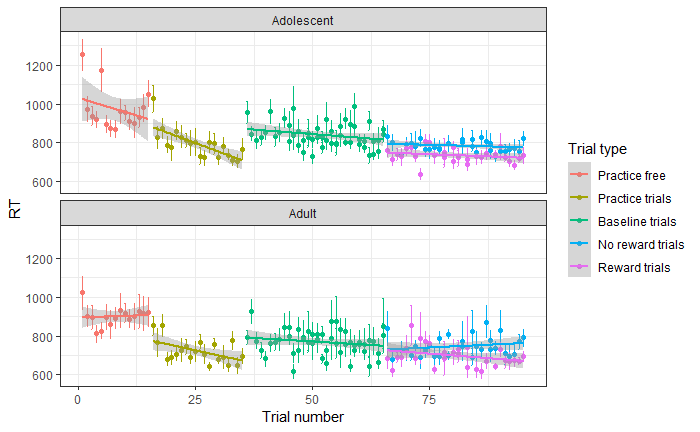


**Figure S1**: Mean reaction time (ms) (± SE) plotted as function of trial number over the course of the practice and test phases of the working memory task. Practice free were the first 15 trials, without a time limit, and Practice trials were the following 20 trials, with participants median RT as a time limit.


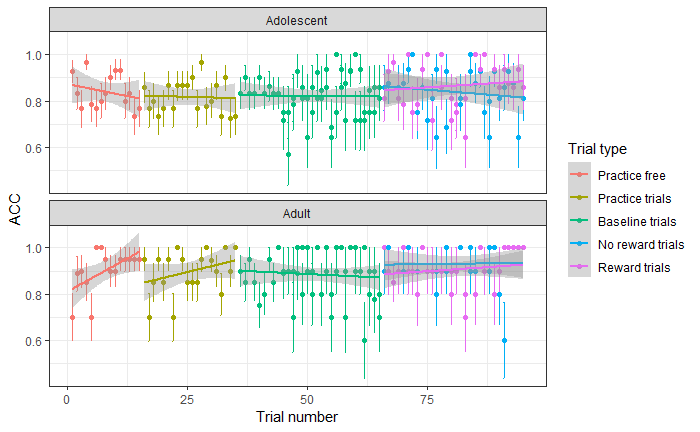


**Figure S2**: Mean accuracy (proportion) (± SE) plotted as function of trial number over the course of the practice and test phases of the working memory task. Practice free were the first 15 trials, without a time limit, and Practice trials were the following 20 trials, with participants median RT as a time limit.
